# Comparative efficacy and safety of restrictive versus liberal transfusion thresholds in anemic preterm infants: a meta-analysis of 12 randomized controlled trials

**DOI:** 10.1007/s00277-022-05072-7

**Published:** 2022-12-21

**Authors:** Xiaoling Fu, Xingdan Zhao, Aihan Weng, Qian Zhang

**Affiliations:** Department of Blood Transfusion, Hainan Women and Children’s Medical Center, Haikou, 570000 Hainan Province China

**Keywords:** Premature infant, Very low birth weight, Extremely low birth weight, Restrictive transfusion, Liberal transfusion

## Abstract

The comparative efficacy and safety of restrictive with liberal transfusion thresholds remain controversial in anemic preterm infants. This meta-analysis aimed to compare the efficacy and safety of these two transfusion thresholds for anemic preterm infants. We searched PubMed, Embase, Cochrane Library, and China National Knowledge Infrastructure (CNKI) for relevant randomized controlled trials (RCTs) comparing restrictive with liberal transfusion thresholds in anemic preterm infants through April 30, 2022. Two independent investigators screened literature, extracted data, and appraised the methodological quality of eligible studies. Meta-analysis was conducted using RevMan version 5.3.5. Twelve RCTs with 4380 preterm infants were included. Liberal transfusion threshold significantly increased the level of hemoglobin after transfusion (mean difference (MD): −10.03; 95% confidence interval (CI): −15.98 to −4.08; *p*=0.001; *I*^2^=94%) and hematocrit (MD: −3.62; 95%CI: −6.78 to −0.46; *p*=0.02; *I*^2^=80%) compared with restrictive transfusion. Infants’ age at first transfusion in restrictive transfusion group was higher than that of infants in liberal transfusion group (MD: 5.08; 95%CI: 2.27 to7.89; *p*=0.004; *I*^2^=54%); however, restrictive transfusion was associated with more time on supplemental oxygen (MD: 3.56; 95%CI: 1.93 to 5.18; *p*<0.001; *I*^2^=62%) and ventilator or CPAP (MD: 3.31; 95%CI: 1.42 to 5.20; *p*=0.006; *I*^2^=75%). For the remaining outcomes, two transfusion strategies were comparable. Furthermore, a series of sensitivity analyses confirmed the robustness of the level of hemoglobin after transfusion, age at first transfusion, time on ventilator or CPAP, and safety outcomes. Evidence with substantial heterogeneity indicates that liberal and restrictive transfusion thresholds are effective and safe blood cell transfusion strategies in anemic preterm infants, but the liberal strategy may be more effective in shortening the length of necessary respiratory support.

**Supplementary Information:**

The online version contains supplementary material available at 10.1007/s00277-022-05072-7.

## Introduction

Preterm infants are at high risk for anemia due to immaturity, impaired erythropoiesis, and frequent blood sampling [1, 2]. Therefore, preterm infants have one of the highest transfusion requirements within the hospital setting [3], especially very (VLBW) or extremely low birth weight (ELBW) infants [4]. More than 90% of ELBW infants received at least one red blood cell transfusion during their initial hospitalization [5, 6]. Red blood cell transfusion has been demonstrated to have several benefits, such as maintaining high hemoglobin [7, 8] and reducing the risks of hypoxemia and apnea of prematurity [9, 10]. Although a low level of hemoglobin was considered to have an adverse impact on growth [11], some studies suggested that red blood cell transfusion can have several complications, such as intraventricular hemorrhage [12, 13], retinopathy of prematurity [14, 15], bronchopulmonary dysplasia [16, 17], necrotizing enterocolitis [18, 19], and death [20]. Therefore, several attentions have been paid to investigating the thresholds for red blood cell transfusion.

Various thresholds for red blood cell transfusion have been applied in clinical practice [21]; however, it could be mainly categorized into two strategies, including restrictive and liberal thresholds [3]. A series of previous randomized controlled trials (RCTs) have investigated the impact of different transfusion thresholds on clinical outcomes of anemic preterm infants but reported conflicting results [22–24]. Subsequently, several meta-analyses [25–28] have further evaluated the comparative efficacy and safety of restrictive versus liberal transfusion thresholds for treating anemic preterm infants and suggested that restrictive transfusion threshold was comparable to liberal transfusion threshold in death and neurodevelopment. However, the value of these findings dedicated to making the clinical decision was limited because several clinically important outcomes were not evaluated in previous meta-analyses, such as age at first transfusion, total transfusion volume, and time on supplemental oxygen. Moreover, some potentially eligible studies [29–32] published in the Chinese language were also not included in the meta-analysis.

Therefore, we conducted this meta-analysis to further evaluate the relative efficacy and safety of restrictive versus liberal transfusion thresholds in anemic preterm infants by including more eligible studies and more comprehensive outcomes.

## Methods

We designed the current meta-analysis following the Cochrane Handbook for Systematic Reviews of Interventions version 6.2 [33]. We reported all results in accordance with the Preferred Reporting Items for Systematic Reviews and Meta-Analyses (PRISMA) statement [34]. It is not necessary to include ethical approval and patients’ informed consent because this is a meta-analysis of published studies. It is necessary to say that the formal protocol of the current meta-analysis was not registered on any platform.

### Search strategy

A systematic search was performed in PubMed, Embase, the Cochrane Library, and China National Knowledge Infrastructure (CNKI) to identify relevant RCTs which compared restrictive with liberal transfusion thresholds in anemic preterm infants. Two independent investigators performed the search. The search was limited from the establishment date of each database until April 30, 2022. We constructed a search strategy using medical subject heading (MeSH) and text words, and the following terms and their analogs were used: “anemia,” “premature infant,” “extremely premature infant,” “blood transfusion,” and “random.” The details of the search strategy are summarized in Table S1. We also manually screened the eligible studies included in the previous meta-analyses and the reference lists of all eligible studies to identify studies missing from database search. Any disagreement regarding study retrieval was resolved by discussion.

### Selection criteria

According to the following selection criteria, two independent investigators selected eligible studies through screening title, abstract, and full text. Specifically speaking, studies were included if (1) participants were determined as anemic preterm infants with low birth weight (LBW, birth weight <2500 g), very low birth weight (VLBW, birth weight <1500 g), or extremely low birth weight (ELBW, birth weight <1000 g); (2) anemic preterm infants were assigned to receive restrictive or liberal transfusion threshold throughout their initial hospitalization; (3) at least one of physiological measurements (the level of hemoglobin after transfusion (g/l) and hematocrit (%)), transfusion-related indicators (age at the first transfusion, transfusion per infant, donor exposure per infant, and total transfusion volume), clinical outcomes (time on supplemental oxygen, time on ventilator or continuous positive airway pressure (CPAP), and the length of hospital stay), and safety outcomes (death or neurodevelopmental impairment, bronchopulmonary dysplasia, necrotizing enterocolitis, any retinopathy of prematurity, retinopathy of prematurity with stage >3, intraventricular hemorrhage, intraventricular hemorrhage with grade >3, apnea, periventricular leukomalacia, and patent ductus arteriosus) had to be reported; and (4) RCTs were published in English with full text.

We excluded ineligible studies according to the exclusion criteria as follows: (1) ineligible study designs such as case series, narrative review, animal study, meta-analysis; (2) essential data were not available for data analysis, and no response was received after sending an e-mail to the corresponding author; and (3) duplicate reports from the same participants that were published by the same group had relatively poor methodological quality and insufficient data.

### Data extraction

Two independent investigators extracted the following essential data from original studies using a pre-designed standard information extraction sheet: characteristics of the included studies (the first author’s name, publication year, country, and sample size), participants’ baseline information (mean gestational age, the percentage of male infants, birth weight, and dose of per transfusion), clinical outcomes, and information of methodological quality. If an eligible study only reported the mean values of each group and *p*-value for the significance test between two groups, we estimated the same standard deviation (SD) for both groups using the formula recommended by the Cochrane Handbook. Moreover, if an eligible study reported results using median with interquartile range (IQR), we transformed it to be mean with SD using the recognized formula [35]. For the level of hemoglobin and hematocrit, we extracted the data after transfusion rather than the changing value between before transfusion and after transfusion for meta-analysis because most studies only reported values at randomization rather than before transfusion. Furthermore, these two values were evenly distributed between the two groups at randomization (level of hemoglobin (g/l): *t*=−0.437, *p*=0.672; hematocrit (%): *t*=0.189, *p*=0.855).

### Risk of bias assessment

We appraised the risk of bias of eligible studies using the Cochrane Collaboration Risk of Bias tool from seven items as follows: random sequence generation (selection bias); allocation concealment (selection bias); blinding of personnel and participants (performance bias): blinding of outcome assessment (detection bias): incomplete outcome data (attrition bias); selective reporting (reporting bias); and other bias sources. Each item was rated as “low,” “unclear,” or “high” risk. Any disagreements regarding the risk of bias assessment were resolved through discussion. The results of the risk of bias assessment were graphically presented using RevMan version 5.4.

### Statistical analysis

Meta-analysis was conducted using the RevMan version 5.4 software (The Cochrane Collaboration, Copenhagen, Denmark). Pooled results were expressed as the mean difference (MD) or relative risk (RR), with a 95% confidence interval (CI) [36]. We evaluated statistical heterogeneity across studies using the chi-square test (Cochrane *Q*) [37] and *I*^2^ statistic [38]. Statistical heterogeneity was significant if the *p*-value <0.1 and *I*^2^ ≥50% [39], and then, sensitivity analysis was introduced to test the robustness of the pooled results. Sensitivity analysis was conducted by using the leave-one-out strategy. Considering the fact that full elimination of variations between studies is impossible, we therefore used the random-effects model to conduct meta-analysis [40, 41]. Nevertheless, we simultaneously calculated all results based on the fixed-effects model to assess the robustness of all results by comparing them with those calculated from the random-effects model. A *p*-value <0.05 was considered statistically significant. In addition, we further evaluated the impact of transfusion thresholds on infants with VLBW and ELBW by excluding infants with LBW. Finally, we used a funnel plot to test the risk of publication bias although the accumulated number of eligible studies did not exceed 10 [42].

## Results

### Study search

**Fig. 1:**
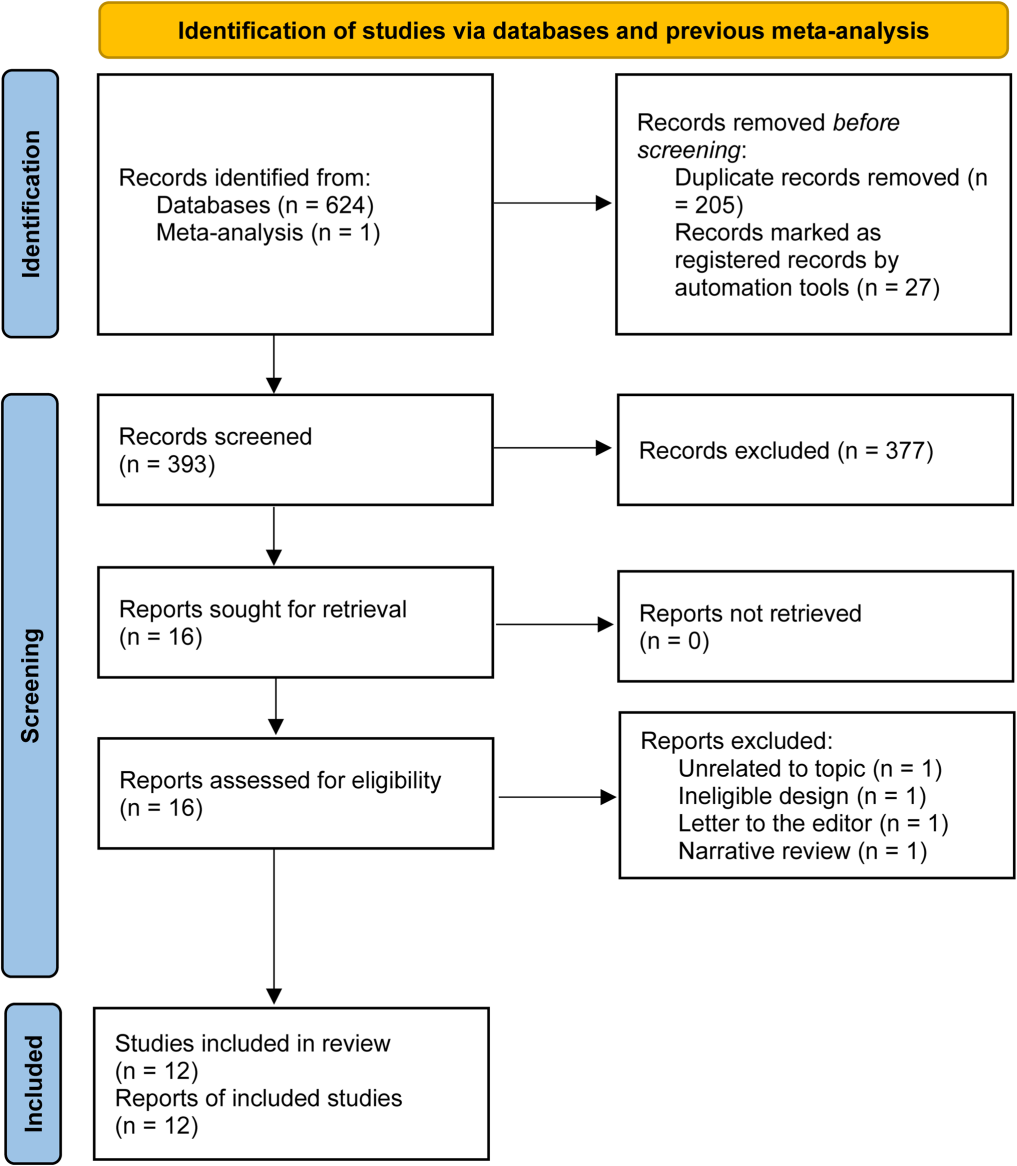
PRISMA flow chart of study retrieval and selection

A total of 624 records were captured from three electronic databases, and 1 study was identified from the previously published meta-analysis. Initially, 205 duplicate records and 27 registered records of clinical trials were automatically removed by EndNote software. Then, 377 unrelated records were excluded through screening their titles and abstracts. After screening full texts of the remaining 16 studies, we continued to exclude 4 ineligible studies due to unrelated to the topic (*n*=1), ineligible study design (*n*=1), letter to the editor (*n*=1), and narrative review (*n*=1). Finally, a total of 12 eligible studies [22–24, 29–32, 43–47] were included in the final analysis. The detailed process of the study retrieval and selection is shown in Fig. 1.

### Characteristics of studies

Among 12 studies included in this meta-analysis, five studies [22, 29–32] were conducted in China, three studies [43, 44, 47] in the USA, three studies [23, 24, 46] in Canada, and one study [45] in Germany. Except for one study [44], other studies were published between 2005 and 2021. The sample size of the individual study ranged from 36 to 1824, with an accumulated number of 4380. Two studies [29, 30] enrolled anemic preterm infants with LBW, five studies [22, 31, 32, 43, 44] enrolled anemic preterm infants with VLBW, and the remaining five studies [23, 24, 45–47] enrolled anemic preterm infants with ELBW. The dose per transfusion of all eligible studies ranged from 10 to 20 ml/kg. Detailed baseline characteristics of 12 eligible studies are presented in Table 1. The transfusion thresholds for two transfusion strategies among all eligible studies are summarized in Table S2.

**Table 1:** Baseline characteristics of 12 eligible studies

Study	Country	Patients	Sample size		Male participants		Gestational age, weeks		Birth weight, g		Mean Hb at randomization, g/dL		Mean hematocrit at randomization, %		Dose of per transfusion
			RG	LG	RG	LG	RG	LG	RG	LG	RG	LG	RG	LG	
Fan et al., 2021 [29]	China	LBW	47	36	22	19	31.76	32.81	1694.47	1915.78	14.3	14.2	43.9	44.3	15 ml/kg
Liu et al., 2012 [30]	China	LBW	47	53	n.r.	n.r.	n.r.	n.r.	<1600	<1600	n.r.	n.r.	n.r.	n.r.	15 ml/kg
Wang et al., 2013 [31]	China	VLBW	44	42	25	24	30.5±1.6	31.1±1.2	1230	1300	n.r.	n.r.	46.3	45.2	15–20 ml/kg
Wu et al., 2017 [32]	China	VLBW	90	90	48	48	30.8±2.2	30.5±2.3	1280.5	1295.5	n.r.	n.r.	46.5	46.5	15–20 ml/kg
Bell et al., 2005 [43]	USA	VLBW	49	51	30	21	27.7±1.7	27.8±2.1	958	954	n.r.	n.r.	50	49	15 ml/kg
Blank et al., 1984 [44]	USA	VLBW	30	26	n.r.	n.r.	29.8±1.8	29.4±2.6	1145.5	1195.6	15.23	16.67	n.r.	n.r.	10 ml/kg
Chen et al., 2009 [22]	China	VLBW	19	17	12	9	29.1±3.0	29.1±2.7	1123.7	1178.2	15.3	15.0	n.r.	n.r.	10 ml/kg
Franz et al., 2020 [45]	Germany	ELBW	521	429	258	246	26.4	26.1	750	745	n.r.	n.r.	46.8	47.3	20 ml/kg
Kirpalani et al., 2006 [46]	Canada	ELBW	223	228	n.r.		<31		<1000		16.4	16.5	n.r.	n.r.	15 ml/kg
Kirpalani et al., 2020 [23]	Canada	ELBW	913	911	419	404	25.9	25.9	757.3	754.9	13.7	13.8	n.r.	n.r.	15 ml/kg
Whyte et al., 2009 [24]	Canada	ELBW	213	208	103	102	26	26	769	769	n.r.	n.r.	n.r.	n.r.	15 ml/kg
Widness et al., 2005 [47]	USA	ELBW	46	47	26	24	26	26	742	734	14.3	14.7	n.r.	n.r.	15 ml/kg

*RG*, restrictive transfusion group; *LG*, liberal transfusion group; *LBW*, low birth weight; *VLBW*, very low birth weight; *ELBW*, extremely low birth weight; *n.r.*, not reported; *n.a.*, not available

### Risk of bias

All eligible studies were regarded as RCTs because the word “random” was mentioned; however, five studies [22, 29–31, 45] did not report the method of generating random sequences. In addition, only four studies [23, 43, 46, 47] reported the methods of concealing allocation. All studies were rated as “low” or “unclear” risk in performance bias, attrition bias, reporting bias, and other bias sources, but two studies were rated as “high” risk in detection bias. Generally, the overall methodological quality of 12 eligible studies is moderate.

### Meta-analysis of physiological measurements

**Fig. 2:**
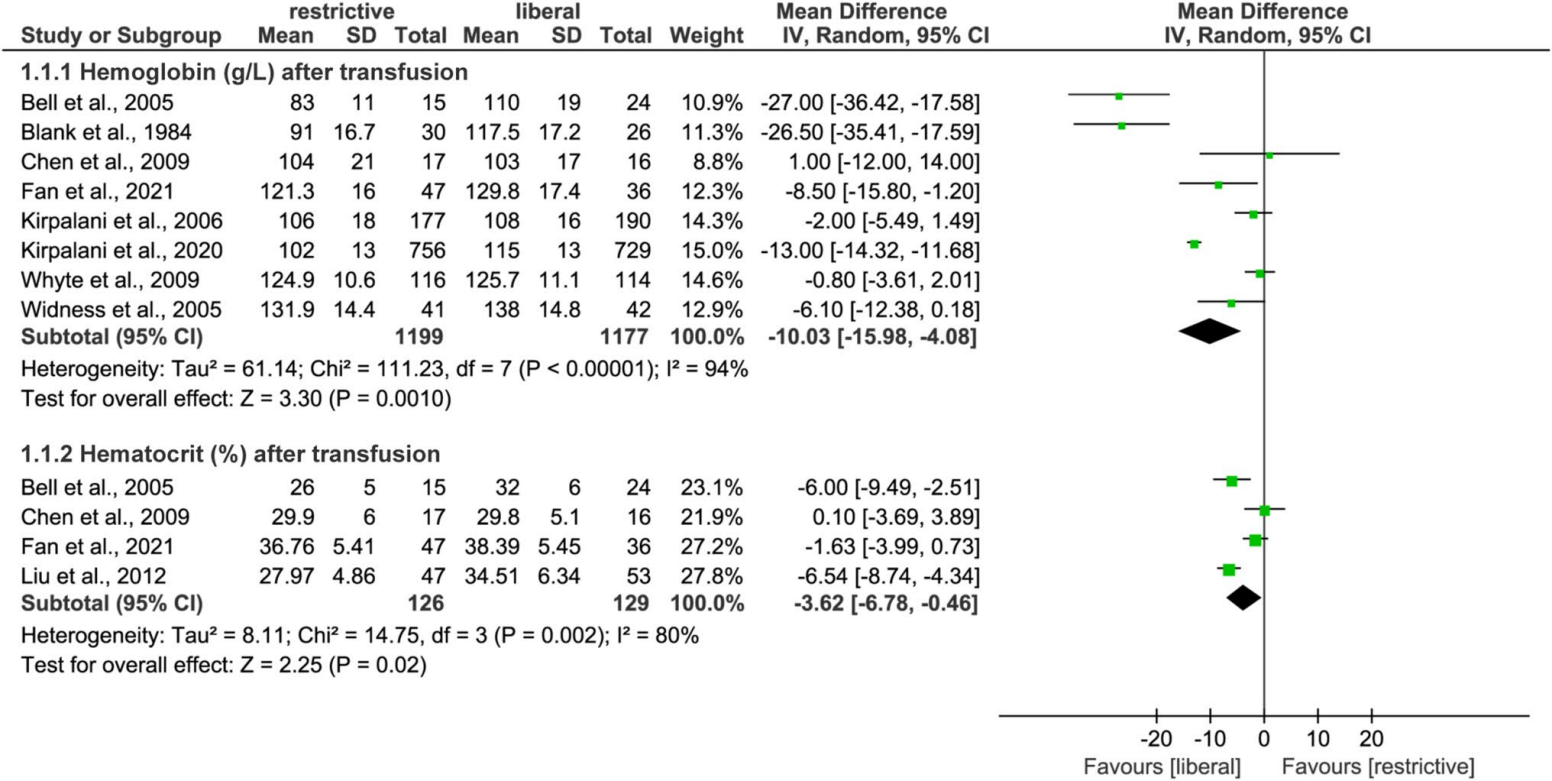
Random-effects meta-analysis of physiological measurements between restrictive and liberal transfusion thresholds. SD, standard difference; IV, inverse variance; CI, confidence interval

A total of 8 studies [22–24, 29, 43, 44, 46, 47] reported the level of hemoglobin after transfusion, and meta-analysis based on the random-effects model suggested that liberal transfusion threshold significantly increased the level of hemoglobin compared to restrictive transfusion threshold (MD: −10.03; 95%CI: −15.98 to −4.08; *p*=0.001). In addition, four studies [22, 29, 30, 43] reported hematocrit after transfusion, and meta-analysis suggested that liberal transfusion threshold was associated with increased hematocrit (MD: −3.62; 95%CI: −6.78 to −0.46; *p*=0.02). The results of physiological measurements are displayed in Fig. 2. The pooled result of hemoglobin (MD: −10.24; 95%CI: −11.34 to −9.14; *p*<0.001) and hematocrit (MD: −3.96; 95%CI: −5.32 to −2.59; *p*<0.001) was not significantly changed by the meta-analyses based on the fixed-effects model (Fig. S2). However, the difference between the two transfusion thresholds in hematocrit changed to be insignificant after excluding LBW infants (MD: −3.00; 95%CI: −8.97 to 2.98; *p*=0.33; Table 2).

**Table 2:** Sensitivity analysis based on exclusion of LBW preterm infants or leave-one-out strategy

Outcomes	Sample size	*I*^2^	Estimate [95%CI]	*p*-value
Hemoglobin, g/l	2376	94%	−10.03 [−15.98, −4.08]	0.001
Exclusion of LBW	2293	95%	−10.27 [−16.82, −3.72]	0.002
Bell et al., 2005 [43]	2337	94%	−7.93 [−14.03, −1.84]	0.01
Blank et al., 1984 [44]	2320	94%	−7.92 [−14.02, −1.83]	0.01
Chen et al., 2009 [22]	2343	94%	−11.09 [−17.32, −4.85]	0.0005
Fan et al., 2021 [29]	2293	95%	−10.27 [−16.82, −3.72]	0.002
Kirpalani et al., 2006 [46]	2009	93%	−11.39 [−18.00, −4.77]	0.0007
Kirpalani et al., 2020 [23]	891	89%	−9.54 [−16.20, −2.89]	0.005
Whyte et al., 2009 [24]	2146	90%	−11.57 [−17.69, −5.45]	0.0002
Widness et al., 2005 [47]	2293	95%	−10.64 [−17.24, −4.04]	0.002
Hematocrit, %	255	80%	−3.62 [−6.78, −0.46]	0.02
Exclusion of LBW	72	81%	−3.00 [−8.97, 2.98]	0.33
Bell et al., 2005 [43]	216	85%	−2.87 [−6.86, 1.12]	0.16
Chen et al., 2009 [22]	222	79%	−4.66 [−8.00, −1.32]	0.006
Fan et al., 2021 [29]	172	78%	−4.35 [−8.20, −0.50]	0.03
Liu et al., 2012 [30]	155	68%	−2.49 [−5.72, 0.73]	0.13
Age at first transfusion, d	305	54%	5.08 [2.27, 7.89]	0.0004
Exclusion of LBW	222	70%	4.47 [0.67, 8.27]	0.02
Bell et al., 2005 [43]	205	68%	4.77 [0.73, 8.81]	0.02
Chen et al., 2009 [22]	269	0%	6.58 [6.05, 7.10]	<0.001
Fan et al., 2021 [29]	222	70%	4.47 [0.67, 8.27]	0.02
Wang et al., 2013 [31]	219	41%	3.75 [−0.20, 7.71]	0.06
Transfusion per infant	2680	97%	−0.48 [−1.59, 0.64]	0.40
Exclusion of LBW	2497	98%	−0.95 [−2.65, 0.76]	0.28
Bell et al., 2005 [43]	2580	97%	−0.24 [−1.41, 0.93]	0.69
Chen et al., 2009 [22]	2644	98%	−0.36 [−1.56, 0.83]	0.55
Fan et al., 2021 [29]	2597	98%	−0.67 [−2.05, 0.70]	0.34
Kirpalani et al., 2006 [46]	2229	97%	−0.43 [−1.66, 0.80]	0.50
Kirpalani et al., 2020 [23]	856	90%	−0.12 [−0.90, 0.65]	0.75
Liu et al., 2012 [30]	2580	98%	−0.67 [−1.98, 0.64]	0.31
Wang et al., 2013 [31]	2594	94%	−0.76 [−1.85, 0.34]	0.18
Donor exposures per infant	551	0%	−0.52 [−0.91, −0.13]	0.0009
Bell et al., 2005 [43]	451	NA	−0.50 [−0.94, −0.06]	0.03
Kirpalani et al., 2006 [46]	100	NA	−0.60 [−1.49, 0.29]	0.18
Total transfusion volume, ml	205	97%	10.83 [−20.82, 42.48]	0.50
Exclusion of LBW	122	98%	10.37 [−37.64, 58.39]	0.67
Chen et al., 2009 [22]	169	91%	23.89 [1.29, 46.49]	0.04
Fan et al., 2021 [29]	122	98%	10.37 [−37.64, 58.39]	0.67
Wang et al., 2013 [31]	119	88%	−1.73 [−27.07, 23.61]	0.89
Time on supplemental oxygen, d	1399	62%	3.56 [1.93, 5.18]	<0.001
Exclusion of LBW	1316	71%	3.41 [1.59, 5.23]	0.0002
Bell et al., 2005 [43]	1299	65%	3.70 [2.14, 5.25]	<0.001
Fan et al., 2021 [29]	1316	71%	3.41 [1.59, 5.23]	0.0002
Franz et al., 2020 [45]	449	0%	4.36 [3.68, 5.04]	<0.001
Wang et al., 2013 [31]	1313	70%	1.87 [−2.18, 5.91]	0.37
Wu et al., 2017 [32]	1219	71%	1.90 [−2.27, 6.07]	0.37
Time on ventilator or CPAP, d	1535	75%	3.31 [1.42, 5.20]	0.0006
Exclusion of LBW	1352	80%	3.58 [0.15, 7.01]	0.04
Bell et al., 2005 [43]	1435	78%	3.45 [1.51, 5.39]	0.0005
Chen et al., 2009 [22]	1499	78%	3.22 [1.30, 5.13]	0.001
Fan et al., 2021 [29]	1452	79%	3.23 [0.55, 5.91]	0.02
Franz et al., 2020 [45]	585	76%	3.77 [1.81, 5.74]	0.0002
Liu et al., 2012 [30]	1435	77%	3.62 [1.40, 5.84]	0.001
Wang et al., 2013 [31]	1449	0%	2.77 [1.93, 3.60]	<0.001
Wu et al., 2017 [32]	1355	79%	3.30 [0.71, 5.88]	0.01
Length of hospital stays, d	3915	59%	−0.86 [−2.84, 1.12]	0.39
Exclusion of LBW	3732	45%	−1.04 [−3.14, 1.05]	0.33
Bell et al., 2005 [43]	3815	63%	−0.83 [−2.89, 1.24]	0.43
Blank et al., 1984 [44]	3859	63%	−0.78 [−2.85, 1.28]	0.46
Chen et al., 2009 [22]	3897	63%	−0.82 [−2.86, 1.21]	0.43
Fan et al., 2021 [29]	3833	49%	−1.51 [−3.29, 0.28]	0.10
Franz et al., 2020 [45]	2916	63%	−0.79 [−2.97, 1.39]	0.48
Kirpalani et al., 2006 [46]	3464	61%	−1.11 [−3.15, 0.93]	0.29
Kirpalani et al., 2020 [23]	2091	60%	−1.11 [−3.28, 1.06]	0.32
Liu et al., 2012 [30]	3815	56%	−0.29 [−2.59, 2.01]	0.80
Wang et al., 2013 [31]	3829	38%	−0.26 [−2.08, 1.56]	0.78
Wu et al., 2017 [32]	3735	54%	−0.95 [−3.33, 1.43]	0.43

*LBW*, low birth weight; *CI*, confidence interval

### Meta-analysis of transfusion-related indicators

**Fig. 3:**
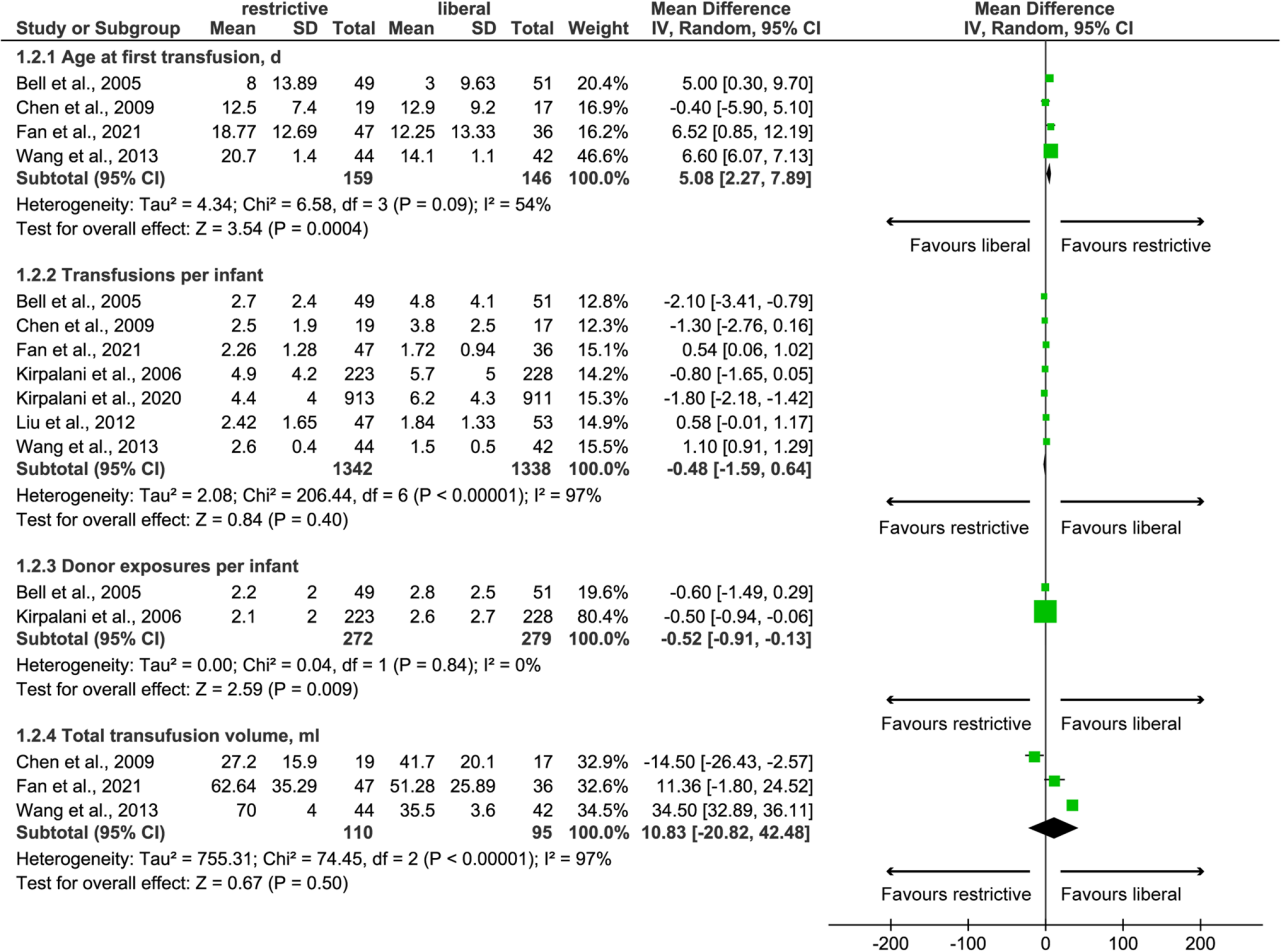
Random-effects meta-analysis of transfusion-related indicators between restrictive and liberal transfusion thresholds. SD, standard difference; IV, inverse variance; CI, confidence interval

Among 12 eligible studies, four [22, 29, 31, 43] and two [43, 46] studies reported age at first transfusion and donor exposure per infant between two groups, respectively. Meta-analysis based on the random-effects model suggested older age at first transfusion (MD: 5.08; 95%CI: 2.27 to 7.89; *p*=0.004) and lower donor exposure (MD: −0.52; 95%CI: −0.91 to −0.13; *p*=0.009) in anemic preterm infants treated by restrictive transfusion threshold (Fig. 3), which was consistent with the pooled results of meta-analyses based on the fixed-effects model (Fig. S3). Transfusion per infant and total transfusion volume were reported by seven [22, 23, 29–31, 43, 46] and three [22, 29, 31] studies, respectively. Meta-analysis based on the random-effects model suggested no statistical difference between restrictive and liberal transfusion thresholds for these two outcomes (Fig. 3); however, meta-analyses based on the fixed-effects model showed that restrictive transfusion threshold was better than liberal transfusion threshold in terms of these two outcomes (Fig. S3). It is noted that the exclusion of LBW preterm infants from the meta-analysis did not change the pooled results (Table 2).

### Meta-analysis of clinical outcomes

**Fig. 4:**
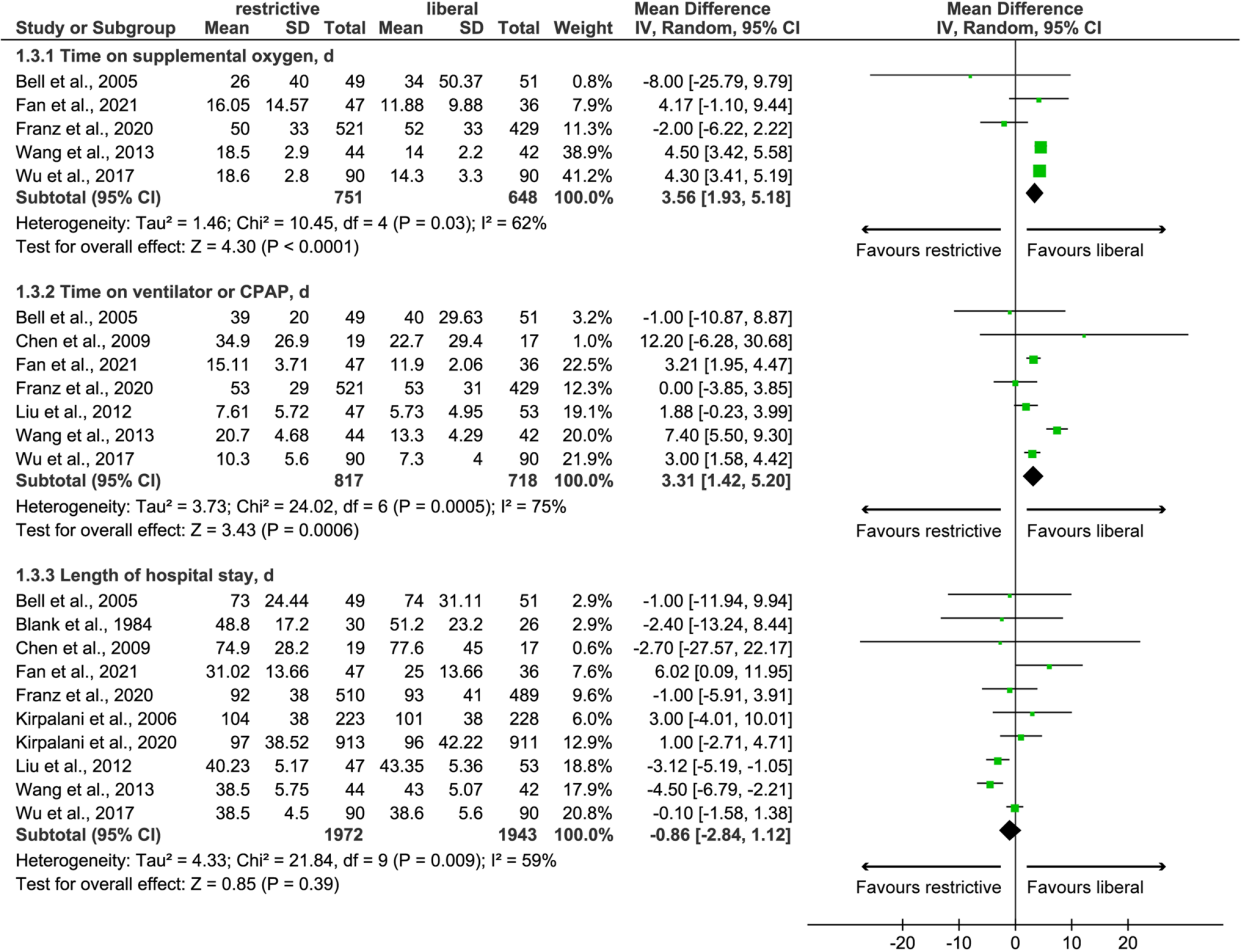
Random-effects meta-analysis of clinical outcomes between restrictive and liberal transfusion thresholds. CPAP, continuous positive airway pressure; SD, standard difference; IV, inverse variance; CI, confidence interval

Five [29, 31, 32, 43, 45], seven [22, 29–32, 43, 45], and ten [22, 23, 29–32, 43–46] studies reported time on supplemental oxygen, time on ventilator or CPAP, and length of hospital stay, respectively. Meta-analysis based on the random-effects model suggested that the restrictive transfusion threshold significantly prolonged the time on supplemental oxygen (MD: 3.56; 95%CI: 1.93 to 5.18; *p*<0.001) and ventilator or CPAP (MD: 3.31; 95%CI: 1.42 to 5.20; *p*=0.006), but had a comparable length of hospital stay between two thresholds (MD: −0.86; 95%CI: −2.84 to 1.12; *p*=0.39), which were not significantly changed after excluding LBW preterm infants (Table 2). Results of clinical outcomes are graphically reported in Fig. 4. However, meta-analyses based on the fixed-effects model showed that restrictive transfusion threshold was associated with significantly shortened length of hospital stay compared to liberal transfusion threshold (Fig. S2).

### Meta-analysis of safety outcomes

A total of 13 safety outcomes were evaluated in this meta-analysis, and meta-analysis based on the random-effects model revealed no statistical difference between restrictive and liberal transfusion groups for any safety outcomes (Table 3), including the composite incidence of death and neurodevelopment impairment (*p*=0.87), overall mortality (*p*=0.91), the incidence of neurodevelopment impairment (*p*=0.34), bronchopulmonary dysplasia (*p*=0.40), necrotizing enterocolitis (*p*=0.36), any retinopathy of prematurity (*p*=0.26), retinopathy of prematurity with stage 3 or above (*p*=0.07), intraventricular hemorrhage (*p*=0.80), intraventricular hemorrhage with grade 3 or above (*p*=0.23), apnea (*p*=0.43), sepsis (*p*=0.81), periventricular leukomalacia (*p*=0.25), and patent ductus arteriosus (*p*=0.05). Furthermore, meta-analysis based on the fixed-effects model yielded consistent results, and results were not significantly reversed after the exclusion of LBW preterm infants (Table 3).

**Table 3:** Pooled results of safety outcomes between restrictive and liberal transfusion thresholds.

Outcomes	Number of studies	*I*^2^	Sample size (RG vs LG)	Model	Estimate [95%CI]	*p*-value
Death or neurodevelopmental impairment	3	7%	1533 vs 1508	REM	1.01 [0.93, 1.09]	0.87
				FEM	1.01 [0.93, 1.09]	0.86
Overall mortality	6	0%	1720 vs 1705	REM	0.99 [0.84, 1.17]	0.91
				FEM	0.99 [0.84, 1.17]	0.91
Exclusion of LBW	5	0%	1673 vs1652	REM	0.99 [0.84, 1.17]	0.92
				FEM	0.99 [0.84, 1.17]	0.93
Neurodevelopment impairment	7	32%	1601 vs 1554	REM	1.08 [0.93, 1.25]	0.34
				FEM	1.04 [0.95, 1.15]	0.41
Exclusion of LBW	6	0%	1554 vs1518	REM	1.02 [0.92, 1.12]	0.76
				FEM	1.02 [0.93, 1.13]	0.65
Bronchopulmonary dysplasia	8	12%	1682 vs 1653	REM	0.96 [0.87, 1.06]	0.40
				FEM	0.96 [0.90, 1.04]	0.32
Exclusion of LBW	6	0%	1588 vs1564	REM	0.95 [0.89, 1.02]	0.17
				FEM	0.95 [0.88, 1.02]	0.18
Necrotizing enterocolitis	7	0%	1809 vs 1780	REM	1.11 [0.89, 1.39]	0.36
				FEM	1.11 [0.89, 1.39]	0.34
Exclusion of LBW	6	0%	1762 vs 1744	REM	1.10 [0.88, 1.38]	0.39
				FEM	1.10 [0.88, 1.38]	0.39
Retinopathy of prematurity	9	0%	1620 vs 1597	REM	0.95 [0.86, 1.04]	0.26
				FEM	0.94 [0.85, 1.04]	0.22
Exclusion of LBW	7	0%	1526 vs 1508	REM	0.95 [0.86, 1.05]	0.29
				FEM	0.94 [0.85, 1.04]	0.26
Retinopathy of prematurity stage >3	5	0%	1543 vs 1511	REM	0.86 [0.74, 1.01]	0.07
				FEM	0.86 [0.73, 1.01]	0.06
Intraventricular hemorrhage	7	39%	816 vs 792	REM	1.04 [0.76, 1.42]	0.80
				FEM	0.99 [0.82, 1.18]	0.90
Exclusion of LBW	6	43%	769 vs 739	REM	1.18 [0.79, 1.76]	0.42
				FEM	1.02 [0.84, 1.24]	0.83
Intraventricular hemorrhage grade 3/4	3	0%	589 vs 560	REM	0.78 [0.53, 1.17]	0.23
				FEM	0.78 [0.53, 1.16]	0.23
Apnea	7	0%	502 vs 507	REM	1.02 [0.97, 1.08]	0.43
				FEM	0.98 [0.89, 1.08]	0.73
Exclusion of LBW	6	0%	455 vs 454	REM	1.02 [0.96, 1.08]	0.46
				FEM	0.97 [0.88, 1.08]	0.62
Sepsis	3	0%	760 vs 737	REM	1.02 [0.88, 1.17]	0.81
				FEM	1.02 [0.89, 1.18]	0.77
Periventricular leukomalacia	6	0%	797 vs 775	REM	1.33 [0.82, 2.15]	0.25
				FEM	1.40 [0.88, 2.22]	0.15
Exclusion of LBW	5	16%	750 vs 722	REM	1.59 [0.64, 3.93]	0.31
				FEM	1.42 [0.88, 2.28]	0.15
Patent ductus arteriosus	5	0%	676 vs 649	REM	0.88 [0.77, 1.00]	0.05
				FEM	0.89 [0.78, 1.01]	0.07

*RG*, restrictive transfusion group; *LG*, liberal transfusion group; *CI*, confidence interval

### Sensitivity analysis

For physiological measurements, transfusion-related indicators, and clinical outcomes, we used the leave-one-out strategy to examine the robustness of pooled results because substantial statistical heterogeneity was detected for all analyses. As presented in Table 2, sensitivity analysis suggested that the pooled result of hematocrit, age at first transfusion, donor exposure per infant, total transfusion volume, and time on supplemental oxygen were significantly changed when leaving one study from meta-analysis each time.

### Publication bias

**Fig. 5:**
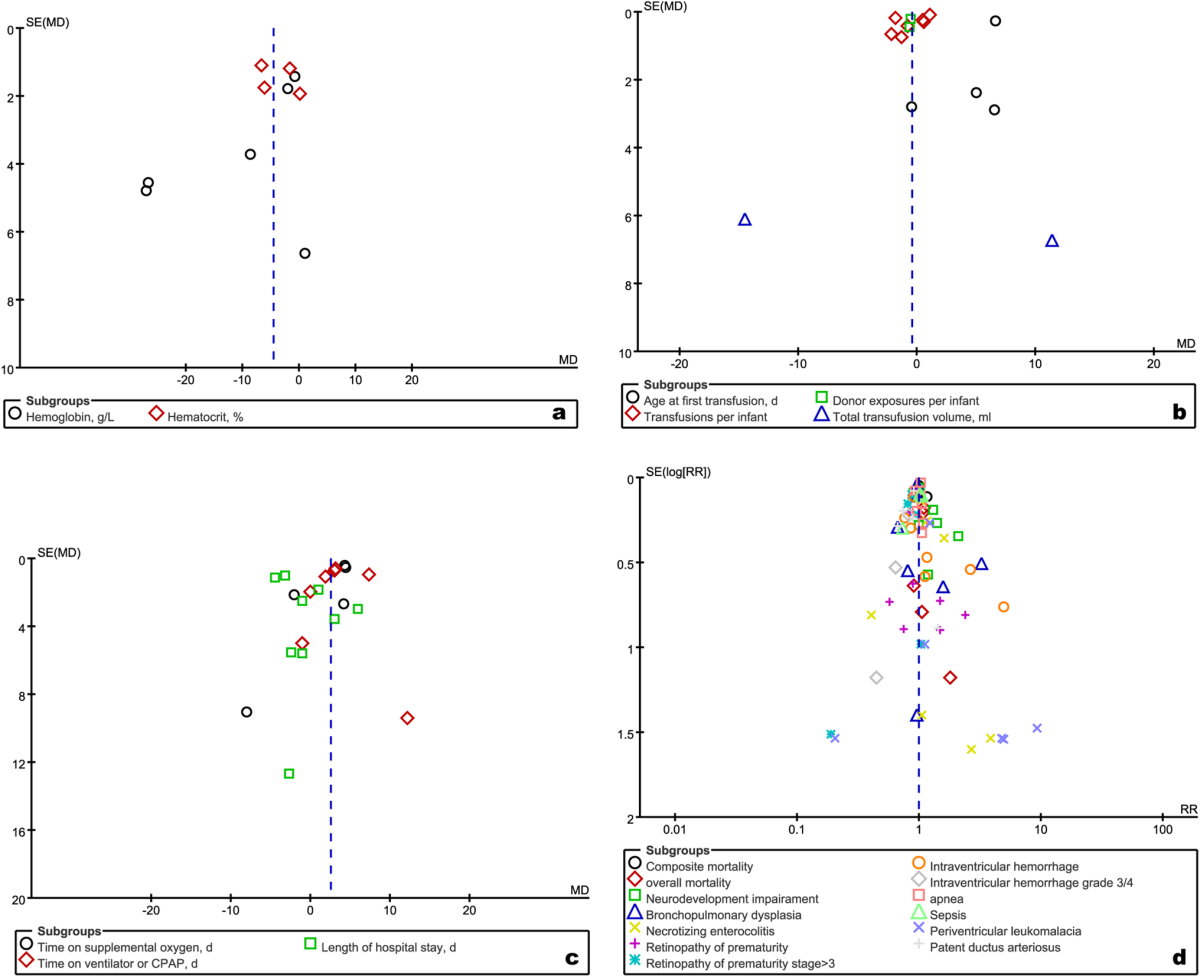
Publication bias plots for the analysis of physiological measurements (**a**), transfusion-related indicators (**b**), clinical outcomes (**c**), and safety outcomes (**d**). SE, standard error; MD, mean difference; RR, relative risk; CPAP, continuous positive airway pressure

We created a funnel plot to evaluate the risk of publication bias for all outcomes qualitatively. As shown in Fig. 5, an asymmetric funnel plot was available for physiological measurements, indicating the risk of publication bias. However, symmetric funnel plots were available for transfusion-related indicators, clinical outcomes, and safety outcomes, suggesting the absence of publication bias for these outcomes.

## Discussion

### Summary of main findings

Red blood cell transfusions are commonly administered to anemic preterm infants, especially to those with VLBW and ELBW. Currently, two transfusion thresholds are available for red blood cell transfusion, including restrictive and liberal transfusion thresholds. Unfortunately, the comparative efficacy and safety of these two transfusion thresholds in anemic preterm infants remain controversial. In the current meta-analysis, a total of 4380 anemic preterm infants with LBW, VLBW, or ELBW were accumulated from 12 eligible studies. Although the comparability of the eligible studies included in this meta-analysis is limited by the high heterogeneity; pooled results remain revealing some promising findings. First, meta-analysis suggested both restrictive and liberal transfusion thresholds may be safe transfusion strategies because they are comparable in terms of safety. Second, liberal transfusion threshold may be more effective in improving the level of hemoglobin after transfusion and decreasing the need for supplemental oxygen, and shortening the time on ventilator or CPAP. Third, the differences in hematocrit, transfusion per infant, total transfusion volume, donor exposure per infant, and the length of hospital stay between the two transfusion thresholds required further validation by larger randomized multicenter studies because the pooled results are significantly changed after excluding LBW anemic preterm infants or changing analysis model from random-effects model to fixed-effects model.

### Comparison with previous meta-analyses

Several meta-analyses [25–28] have compared the efficacy and safety of restrictive versus liberal transfusion thresholds. In 2014, Ibrahim et al. [25] firstly investigated the comparative effects of restrictive with liberal transfusion thresholds on clinical outcomes in VLBW infants, in which three studies involving 625 preterm infants were included. Finally, the authors found that restrictive transfusion was associated with less transfusion and donor exposure per infant, which were inconsistent with our findings. It is noted that limited eligible studies greatly impaired the robustness and reliability of the findings of this meta-analysis. Since then, in 2021, Wang and colleagues performed a systematic review and meta-analysis to further assess the efficacy and safety of restrictive versus liberal transfusion thresholds in VLBW infants [27]. It is noted that this meta-analysis included six studies with 3483 infants to evaluate more comprehensive outcomes, such as the level of hemoglobin after treatment and the composite incidence of death and neurodevelopment impairment, and reported no difference between restrictive and liberal transfusion thresholds for all target outcomes except for the level of hemoglobin, which were consistent with our findings. Unfortunately, other clinically important outcomes were not considered in this meta-analysis, such as transfusion-related indicators and time on ventilator or CPAP. In 2022, two meta-analyses [26, 28] published in the Chinese language also investigated the safety of restrictive versus liberal transfusion thresholds in VLBW infants. These two meta-analyses reported consistent results with our meta-analysis; however, the inclusion of limited eligible studies significantly decreased its reliability.

Compared with previous meta-analyses, the current study has three strengths. The methodology and data of this study are different from those of previous meta-analysis. First, we searched Chinese database to include more eligible studies in the final analysis, which benefits to increase the statistical power and then generate more robust and reliable findings. Second, we used random-effects model to conservatively estimate all results and used leave-one-out strategy to further exam the robustness of pooled results. Third, more comprehensive outcomes were considered in the current meta-analysis such as total transfusion volume, time on supplemental oxygen, and time on ventilator or CPAP. Therefore, results from this meta-analysis benefit to evaluate the efficacy and safety of restrictive and liberal transfusion thresholds more systematically.

### Explanation of main findings

Red blood cell transfusions are commonly used to treat low hemoglobin levels in anemic preterm infants; however, a previous meta-analysis has described a correlation between red blood cell transfusion and the occurrence of retinopathy of prematurity in premature infants [48]. In the present meta-analysis, we did not find significant difference in the incidence of retinopathy of prematurity between two transfusion thresholds because transfusion per infant and total transfusion volume were comparable in both groups.

The proper use of oxygen may be lifesaving; however, excessive supplemental oxygen in preterm infants is a risk factor of retinopathy of prematurity, bronchopulmonary dysplasia, and longer hospital stay [49]. The current meta-analysis revealed that restrictive transfusion threshold was associated with prolonged time on supplemental oxygen in preterm infants; however, no difference was detected in the occurrence of retinopathy of prematurity and bronchopulmonary dysplasia and hospital stay. Studies indicated that if higher-grade retinopathy of prematurity is already present, the administration of oxygen to prevent relative retinal hypoxia while maintaining stage-dependent narrow oxygen saturation limits may minimize retinopathy of prematurity proliferation [50]. It is noted that eligible studies included in the present meta-analysis reported a wide range of tolerated Hb/Hct levels after transfusion, which might answer why the difference between the two strategies in the occurrence of retinopathy of prematurity and bronchopulmonary dysplasia does not achieve statistical significance. Moreover, sensitivity analysis significantly changed the result of time on supplemental oxygen; therefore, future studies are warranted to further evaluate this outcome.

In addition, iron deficiency [51, 52] has been demonstrated as an important risk factor for neurodevelopment; however, the incidence of neurodevelopment between restrictive and liberal transfusion thresholds was not significantly different although liberal transfusion kept significantly higher level of hemoglobin. Interestingly, evidence suggested that erythropoietin failed to improve neurodevelopmental outcomes despite the increase in red blood cells and hemoglobin concentration [53].

### Limitations

Compared to previous meta-analyses, the current study has several strengths mentioned above, but several limitations should also be further explained. First, significant statistical heterogeneity was detected for physiological measurements, transfusion-related indicators, and clinical outcomes, and the level was not greatly decreased although sensitivity analysis with the leave-one-out strategy was conducted. Therefore, we could not eliminate the negative impact of variations in patient variables on our findings. Therefore, future study should especially ensure the homogeneity of the participants as much as possible. Second, there were differences across studies in birth weight; however, we designed two strategies to examine the robustness of pooled results, including the exclusion of LBW infants and the leave-one-out method (see Tables 2 and 3). Third, we detected the risk of publication bias for physiological measurements, which might have a negative impact on the pooled results. Certainly, we did not use the quantitative method (Egger and Begg tests) to test the symmetry of the funnel plot. Therefore, we could not eliminate the error of visual inspection. Fourth, there were differences across studies in the definition of thresholds (see Table S2); however, we did not conduct subgroup analysis to investigate the impact of different transfusion thresholds on outcomes because this strategy will further decrease the number of eligible studies included in the individual analysis, thereby greatly reducing the statistical power. Future studies may use the network meta-analysis to determine the comparative efficacy and safety of different transfusion thresholds in preterm infants when sufficient studies are published. Fifth, there are a limited number of eligible studies for certain outcomes, such as donor exposures per infant and total transfusion volume, so pooled results should be interpreted with caution due to insufficient statistical power. Sixth, we were unable to calculate concrete increases in hemoglobin and hematocrit values in individual studies because only two studies reported values before transfusion.

## Conclusion

Liberal transfusion threshold significantly increases the level of hemoglobin and is associated with a shorter time on ventilator or CPAP in preterm infants. Moreover, liberal transfusion threshold most likely increases hematocrit and shortened the time on supplemental oxygen. Two transfusion thresholds were comparable for all safety outcomes. Overall, both liberal and restrictive transfusion thresholds are effective and safer strategies to red blood cell transfusion in anemic preterm infants, but liberal strategy is more effective in shortening the length of necessary respiratory support. Given the limitations of this meta-analysis, we suggest that more studies to validate our findings, especially the differences between the two transfusion thresholds in terms of hematocrit, transfusion per infant, total transfusion volume, donor exposure per infant, and the length of hospital stay.

## Supplementary information

Figure S1. Risk of bias summary (a) and graph (b). (PNG 729 kb)

High Resolution Image (TIFF 71473 kb)

Figure S2. Fixed-effects meta-analysis of physiological measurements between restrictive and liberal transfusion thresholds. SD, standard difference; IV, inverse variance; CI, confidence interval. (PNG 766 kb)

High Resolution Image (TIFF 182383 kb)

Figure S3. Fixed-effects meta-analysis of transfusion-related indicators between restrictive and liberal transfusion thresholds. SD, standard difference; IV, inverse variance; CI, confidence interval. (PNG 1136 kb)

High Resolution Image (TIFF 265126 kb)

Figure S4. Fixed-effects meta-analysis of clinical outcomes between restrictive and liberal transfusion thresholds. CPAP, continuous positive airway pressure; SD, standard difference; IV, inverse variance; CI, confidence interval. (PNG 1270 kb)

High Resolution Image (TIFF 276749 kb)

Table S1. Detailed search strategy. (DOCX 21 kb)

Table S2. Transfusion thresholds for two transfusion strategies among all eligible studies. (DOCX 18 kb)

## Data Availability

The datasets generated during and/or analyzed during the current study are available from the corresponding author on reasonable request.
