# Supplementary figures and images for: Comparative efficacy and safety of restrictive versus liberal transfusion thresholds in anemic preterm infants: a meta-analysis of 12 randomized controlled trials
Source: Ann Hematol. 2022 Dec 21;102(2):283–97. doi: 10.1007/s00277-022-05072-7 (PMC9889497; doi:10.1007/s00277-022-05072-7)

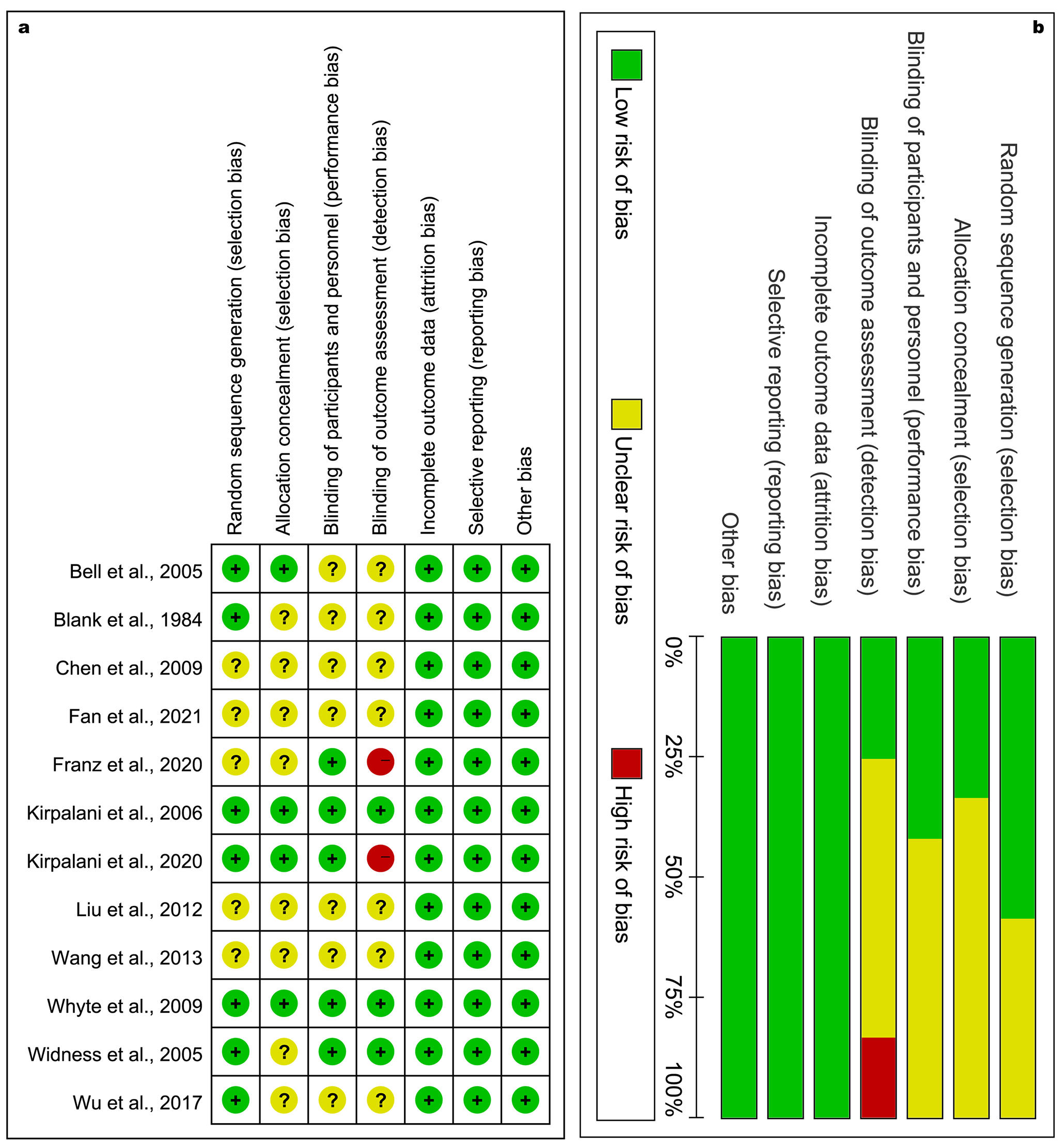

Supplement: Supplementary file 1 — Risk of bias summary (a) and graph (b). (PNG 729 kb) [file 277_2022_5072_Fig6_ESM.png]

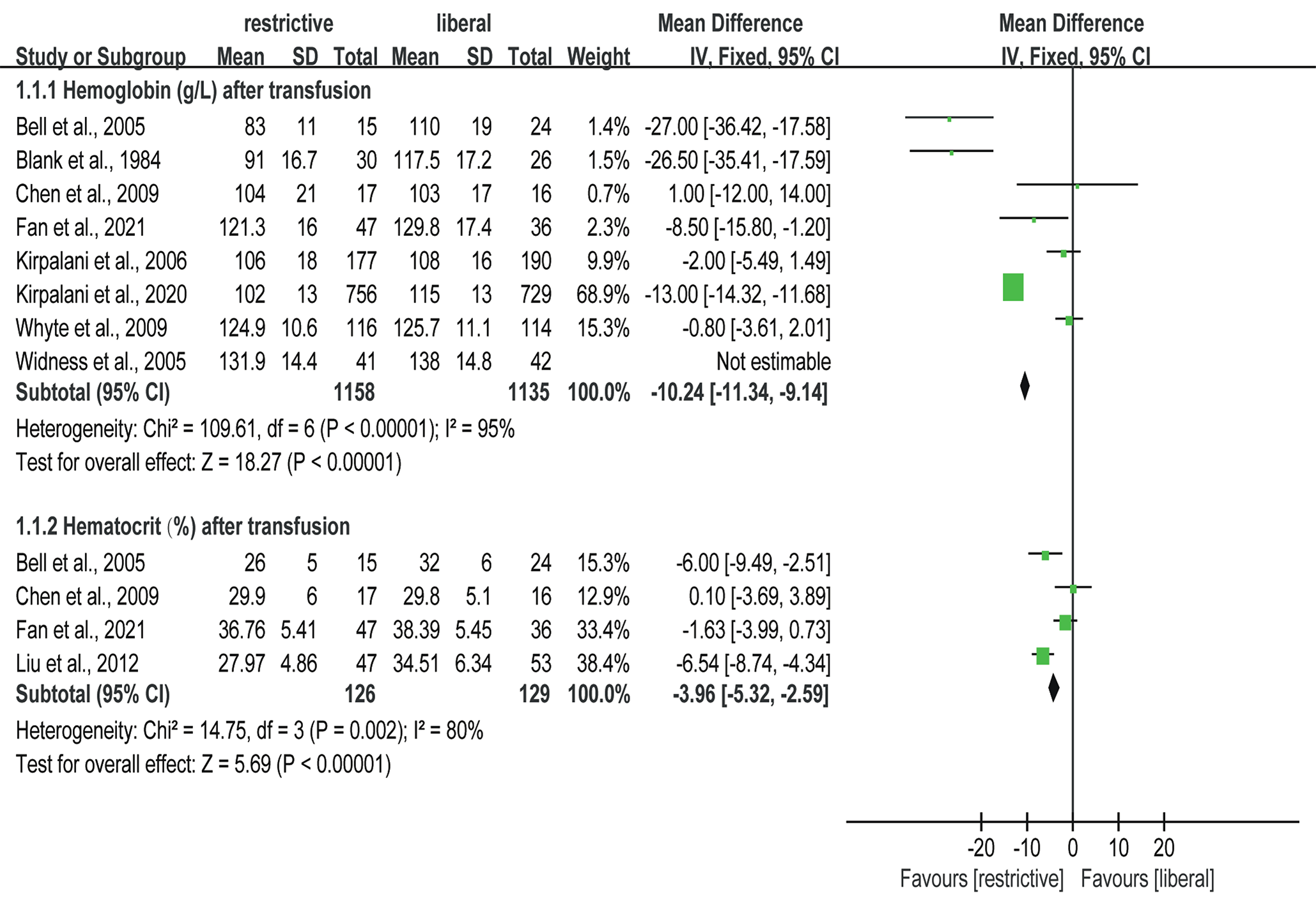

Supplement: Supplementary file 3 — Fixed-effects meta-analysis of physiological measurements between restrictive and liberal transfusion thresholds. SD, standard difference; IV, inverse variance; CI, confidence interval. (PNG 766 kb) [file 277_2022_5072_Fig7_ESM.png]

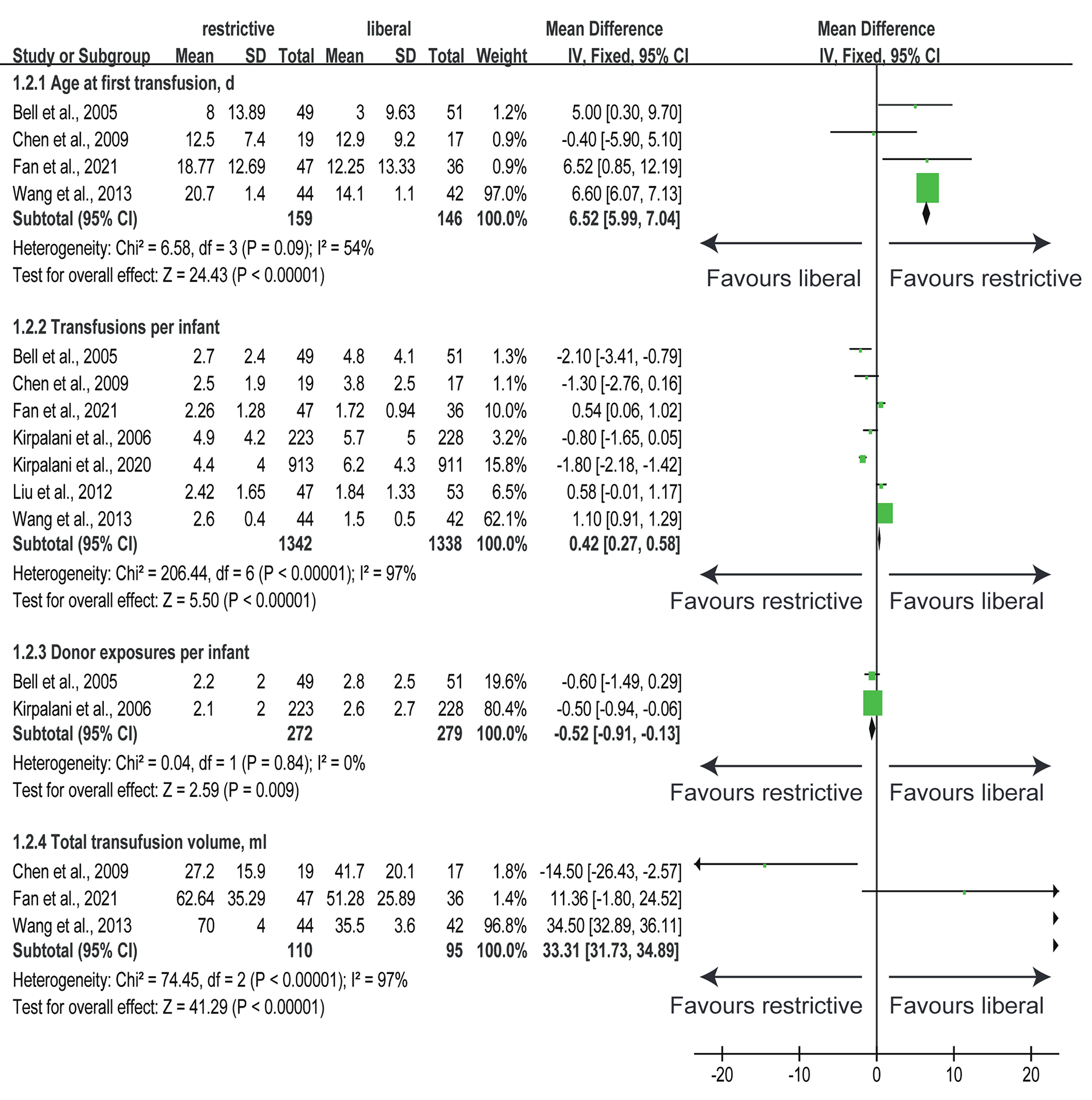

Supplement: Supplementary file 5 — Fixed-effects meta-analysis of transfusion-related indicators between restrictive and liberal transfusion thresholds. SD, standard difference; IV, inverse variance; CI, confidence interval. (PNG 1136 kb) [file 277_2022_5072_Fig8_ESM.png]

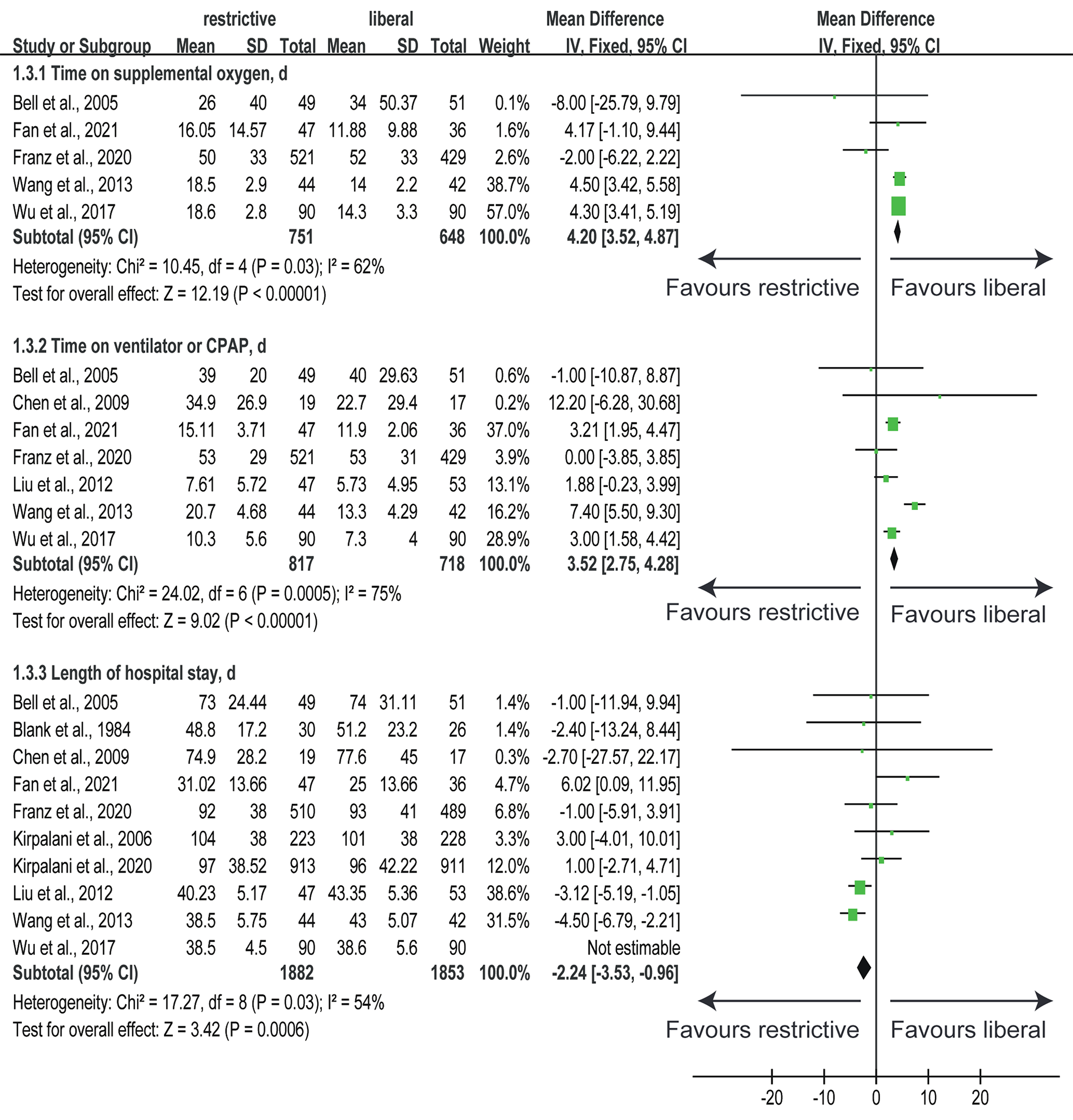

Supplement: Supplementary file 7 — Fixed-effects meta-analysis of clinical outcomes between restrictive and liberal transfusion thresholds. CPAP, continuous positive airway pressure; SD, standard difference; IV, inverse variance; CI, confidence interval. (PNG 1270 kb) [file 277_2022_5072_Fig9_ESM.png]
