# Supplementary material for: Comparative efficacy and safety of restrictive versus liberal transfusion thresholds in anemic preterm infants: a meta-analysis of 12 randomized controlled trials
Source: Ann Hematol. 2022 Dec 21;102(2):283–97. doi: 10.1007/s00277-022-05072-7 (PMC9889497; doi:10.1007/s00277-022-05072-7)
Supplement: Supplementary file 9 — Detailed search strategy. (DOCX 21 kb) [file 277_2022_5072_MOESM5_ESM.docx]

**Comparative efficacy and safety of restrictive versus liberal transfusion thresholds in anemic preterm infants: a meta-analysis of 12 randomized controlled trials**

**Running title: Transfusion thresholds in preterm infants**

**Journal: ANNALS OF HEMATOLOGY**

Xiaoling Fu^*^, Xingdan Zhao, Aihan Weng, Qian Zhang

Department of Blood Transfusion, Hainan Women and Children’s Medical Center, Haikou, 570000, Hainan Province, China.

**^*^Correspondence to:**

Xiaoling Fu

Department of Blood Transfusion, Hainan Women and Children’s Medical Center, Haikou, 570000, Hainan Province, China.

Phone number: 18689823522

E-mail: fuxiaoling88@126.com

PubMed

| No. | Search Details | Results |
| --- | --- | --- |
| 1 | "Anemia"[MeSH Terms] | 170,766 |
| 2 | "anemia"[Title/Abstract] | 128,546 |
| 3 | "Anemia"[MeSH Terms] OR "Anemia"[Title/Abstract] | 226,400 |
| 4 | "infant, extremely premature"[MeSH Terms] OR "infant, premature"[MeSH Terms] | 62,002 |
| 5 | "extremely premature infant"[Title/Abstract] OR "extremely preterm infants"[Title/Abstract] OR "extremely preterm infant"[Title/Abstract] OR "extremely premature infants"[Title/Abstract] OR "premature infant"[Title/Abstract] OR "preterm infants"[Title/Abstract] OR "preterm infant"[Title/Abstract] OR "premature infants"[Title/Abstract] OR "neonatal prematurity"[Title/Abstract] | 47,943 |
| 6 | "infant, extremely premature"[MeSH Terms] OR "infant, premature"[MeSH Terms] OR "extremely premature infant"[Title/Abstract] OR "extremely preterm infants"[Title/Abstract] OR "extremely preterm infant"[Title/Abstract] OR "extremely premature infants"[Title/Abstract] OR "premature infant"[Title/Abstract] OR "preterm infants"[Title/Abstract] OR "preterm infant"[Title/Abstract] OR "premature infants"[Title/Abstract] OR "neonatal prematurity"[Title/Abstract] | 79,820 |
| 7 | "Blood Transfusion"[MeSH Terms] | 90,574 |
| 8 | "transfusion"[Title/Abstract] OR "Blood Transfusion"[MeSH Terms] | 150,532 |
| 9 | "random*"[All Fields] OR "allocation*"[All Fields] OR "placeboes"[All Fields] OR "placebos"[MeSH Terms] OR "placebos"[All Fields] OR "placebo"[All Fields] | 1,684,009 |
| 10 | ("Anemia"[MeSH Terms] OR "Anemia"[Title/Abstract]) AND ("infant, extremely premature"[MeSH Terms] OR "infant, premature"[MeSH Terms] OR ("extremely premature infant"[Title/Abstract] OR "extremely preterm infants"[Title/Abstract] OR "extremely preterm infant"[Title/Abstract] OR "extremely premature infants"[Title/Abstract] OR "premature infant"[Title/Abstract] OR "preterm infants"[Title/Abstract] OR "preterm infant"[Title/Abstract] OR "premature infants"[Title/Abstract] OR "neonatal prematurity"[Title/Abstract])) AND ("transfusion"[Title/Abstract] OR "Blood Transfusion"[MeSH Terms]) AND ("random*"[All Fields] OR "allocation*"[All Fields] OR ("placeboes"[All Fields] OR "placebos"[MeSH Terms] OR "placebos"[All Fields] OR "placebo"[All Fields])) | 128 |
| 11 | (("Anemia"[MeSH Terms] OR "Anemia"[Title/Abstract]) AND ("infant, extremely premature"[MeSH Terms] OR "infant, premature"[MeSH Terms] OR ("extremely premature infant"[Title/Abstract] OR "extremely preterm infants"[Title/Abstract] OR "extremely preterm infant"[Title/Abstract] OR "extremely premature infants"[Title/Abstract] OR "premature infant"[Title/Abstract] OR "preterm infants"[Title/Abstract] OR "preterm infant"[Title/Abstract] OR "premature infants"[Title/Abstract] OR "neonatal prematurity"[Title/Abstract])) AND ("transfusion"[Title/Abstract] OR "Blood Transfusion"[MeSH Terms]) AND ("random*"[All Fields] OR "allocation*"[All Fields] OR ("placeboes"[All Fields] OR "placebos"[MeSH Terms] OR "placebos"[All Fields] OR "placebo"[All Fields]))) AND (humans[Filter]) | 124 |

EMBASE

| No. | Query | Results |
| --- | --- | --- |
| #1 | anemia:ti,ab,kw | 195363 |
| #2 | 'anemia'/exp | 445316 |
| #3 | #1 OR #2 | 477187 |
| #4 | 'extremely premature infant':ti,ab,kw OR 'extremely preterm infants':ti,ab,kw OR 'extremely preterm infant[':ti,ab,kw OR 'extremely premature infants':ti,ab,kw OR 'premature infant':ti,ab,kw OR 'preterm infants':ti,ab,kw OR 'preterm infant':ti,ab,kw OR 'premature infants':ti,ab,kw OR 'neonatal prematurity':ti,ab,kw | 61090 |
| #5 | 'prematurity'/exp | 124873 |
| #6 | #4 OR #5 | 136986 |
| #7 | transfusion:ti,ab,kw | 172325 |
| #8 | 'blood transfusion'/exp | 213000 |
| #9 | #7 OR #8 | 278926 |
| #10 | random*:ti,ab,kw OR allocation:ti,ab,kw OR placebo:ti,ab,kw | 1960522 |
| #11 | #3 AND #6 AND #9 AND #10 | 196 |
| #12 | #11 AND [embase]/lim | 174 |
| #13 | #11 AND [embase]/lim AND 'human'/de | 169 |

Cochrane Registry for Controlled Trials (CENTRAL)

| No. | Query | Results |
| --- | --- | --- |
| #1 | (anemia):ti,ab,kw | 22146 |
| #2 | MeSH descriptor: [Anemia] explode all trees | 5715 |
| #3 | #1 or #2 | 22613 |
| #4 | (Extremely Premature Infant):ti,ab,kw OR (Extremely Preterm Infants):ti,ab,kw OR (Extremely Preterm Infant):ti,ab,kw OR (Extremely Premature Infants):ti,ab,kw OR (Premature Infant):ti,ab,kw | 8384 |
| #5 | (Preterm Infants):ti,ab,kw OR (Preterm Infant):ti,ab,kw OR (Premature Infants):ti,ab,kw OR (Neonatal Prematurity):ti,ab,kw | 14389 |
| #6 | #4 or #5 | 15151 |
| #7 | MeSH descriptor: [Infant, Extremely Premature] explode all trees | 247 |
| #8 | MeSH descriptor: [Infant, Premature] explode all trees | 4189 |
| #9 | #6 or #7 or #8 | 15151 |
| #10 | (transfusion):ti,ab,kw | 16415 |
| #11 | MeSH descriptor: [Blood Transfusion] explode all trees | 3744 |
| #12 | #10 or #11 | 16626 |
| #13 | #3 and #9 and #12 in Trials | 181 |
