# Supplementary material for: Comparative efficacy and safety of restrictive versus liberal transfusion thresholds in anemic preterm infants: a meta-analysis of 12 randomized controlled trials
Source: Ann Hematol. 2022 Dec 21;102(2):283–97. doi: 10.1007/s00277-022-05072-7 (PMC9889497; doi:10.1007/s00277-022-05072-7)
Supplement: Supplementary file 10 — Transfusion thresholds for two transfusion strategies among all eligible studies. (DOCX 18 kb) [file 277_2022_5072_MOESM6_ESM.docx]

**Comparative efficacy and safety of restrictive versus liberal transfusion thresholds in anemic preterm infants: a meta-analysis of 12 randomized controlled trials**

**Running title: Transfusion thresholds in preterm infants**

**Journal: ANNALS OF HEMATOLOGY**

Xiaoling Fu^*^, Xingdan Zhao, Aihan Weng, Qian Zhang

Department of Blood Transfusion, Hainan Women and Children’s Medical Center, Haikou, 570000, Hainan Province, China.

**^*^Correspondence to:**

Xiaoling Fu

Department of Blood Transfusion, Hainan Women and Children’s Medical Center, Haikou, 570000, Hainan Province, China.

Phone number: 18689823522

E-mail: fuxiaoling88@126.com

**Table S2.** Transfusion thresholds for two transfusion strategies among all eligible studies.

| Study | RG | LG |
| --- | --- | --- |
| Fan et al., 2021 | Mechanically ventilated to maintain FiO2 >40%: Hct ≤35% or Hb ≤110g/L  Mechanically ventilated to maintain FiO2 <40%: Hct ≤30% or Hb ≤100g/L  Anemia or increased need for oxygen without respiratory support: Hct ≤25% or Hb ≤80g/L | Thresholds increased from transfusion thresholds used in the restrictive transfusion group were applied to the transfusion liberal group but definitive transfusion thresholds were reported in this group. |
| Liu et al., 2012 | Neither positive pressure nor oxygen: Hct <21%  Oxygen or respiratory support due to apnea: Hct >31%  CPAP or supplemental oxygen: Hct >36% | Neither positive pressure nor oxygen: Hct <30%  Oxygen or respiratory support due to apnea: Hct >38%  CPAP or supplemental oxygen: Hct >46% |
| Wang et al., 2013 | Neither positive pressure nor oxygen: Hct ≤25%  Mechanically ventilated: Hct ≤36%  CPAP or supplemental oxygen: Hct ≤31% | Neither positive pressure nor oxygen: Hct >25%  Mechanically ventilated: Hct >36%  CPAP or supplemental oxygen: Hct >31% |
| Wu et al., 2017 | Hb <70 g/L | Hb <100 g/L |
| Bell et al., 2005 | Neither positive pressure nor oxygen: Hct ≤22%  Mechanically ventilated: Hct <34%  CPAP or supplemental oxygen: Hct ≤28% | Neither positive pressure nor oxygen: Hct ≤30%  Mechanically ventilated: Hct <46%  CPAP or supplemental oxygen: Hct ≤38% |
| Blank et al., 1984 | Preoperative surgery Hb level of <10 g/dL, tachycardia (>170 bpm for 4 days), no weight gain for 7 days (140 calories/kg/d), and apnea not responsive to theophylline. | Transfusion to maintain Hb >10 g/dL |
| Chen et al., 2009 | Assisted ventilation: Hct >35%  CPAP: Hct >30%  Spontaneous breath: Hct >22% | Assisted ventilation: Hct >45%  CPAP: Hct >40%  Spontaneous breath: Hct >30% |
| Franz et al., 2020 | Age: from randomization to 7 days Critical heath state: Hct <34% Noncritical heath state: Hct <28% Age: 8-21 days  Critical heath state: Hct <30%  Noncritical heath state: Hct <24% Age: >21 days Critical heath state: Hct <27%  Noncritical heath state: Hct <21% | Age: from randomization to 7 days Critical heath state: Hct <41% Noncritical heath state: Hct <35% Age: 8-21 days  Critical heath state: Hct <37%  Noncritical heath state: Hct <31% Age: >21 days Critical heath state: Hct <34%  Noncritical heath state: Hct <28% |
| Kirpalani et al., 2006 | Age: 1-7 days respiratory support: Hb <11.5 g/dl (ca); Hb <10.4 g/dl (ce) no respiratory support: Hb <10.0 g/dl (ca); Hb <9.0 g/dl (ce) Age: 8-14 days respiratory support: Hb <10.0 g/dl (ca); Hb <9.0 g/dl (ce) no respiratory support: Hb <85.0 g/dl (ca); Hb < 7.7 g/dl (ce) Age: >15 days respiratory support: Hb <8.50 g/dl (ca); Hb <7.7 g/dl (ce) no respiratory support: Hb <7.5 g/dl (ca); Hb <68 g/dl (ce) | Age: 1-7 days respiratory support: Hb <13.5 g/dl (ca); Hb <12.2 g/dl (ce) no respiratory support: Hb <12.0 g/dl (ca); Hb <10.9 g/dl (ce) Age: 8-14 days respiratory support: Hb <12.0 g/dl (ca); Hb <10.9 g/dl (ce) no respiratory support: Hb <10.0 g/dl (ca); Hb <9.0 g/dl (ce) Age: >15 days respiratory support: Hb <10.0 g/dl (ca); Hb <9.0 g/dl (ce) no respiratory support: Hb <8.5 g/dl (ca); Hb <7.7 g/dl (ce) |
| Kirpalani et al., 2020 | Age: 1-7 days respiratory support: Hb <11.5 g/dl (ca); Hb <10.4 g/dl (ce) no respiratory support: Hb <10.0 g/dl (ca); Hb <9.0 g/dl (ce) Age: 8-14 days respiratory support: Hb <10.0 g/dl (ca); Hb <9.0 g/dl (ce) no respiratory support: Hb <85.0 g/dl (ca); Hb < 7.7 g/dl (ce) Age: >15 days respiratory support: Hb <8.50 g/dl (ca); Hb <7.7 g/dl (ce) no respiratory support: Hb <7.5 g/dl (ca); Hb <68 g/dl (ce) | Age: 1-7 days respiratory support: Hb <13.5 g/dl (ca); Hb <12.2 g/dl (ce) no respiratory support: Hb <12.0 g/dl (ca); Hb <10.9 g/dl (ce) Age: 8-14 days respiratory support: Hb <12.0 g/dl (ca); Hb <10.9 g/dl (ce) no respiratory support: Hb <10.0 g/dl (ca); Hb <9.0 g/dl (ce) Age: >15 days respiratory support: Hb <10.0 g/dl (ca); Hb <9.0 g/dl (ce) no respiratory support: Hb <8.5 g/dl (ca); Hb <7.7 g/dl (ce) |
| Whyte et al., 2009 | Age: 1-7 days respiratory support: Hb <11.5 g/dl (ca); Hb <10.4 g/dl (ce) no respiratory support: Hb <10.0 g/dl (ca); Hb <9.0 g/dl (ce) Age: 8-14 days respiratory support: Hb <10.0 g/dl (ca); Hb <9.0 g/dl (ce) no respiratory support: Hb <85.0 g/dl (ca); Hb < 7.7 g/dl (ce) Age: >15 days respiratory support: Hb <8.50 g/dl (ca); Hb <7.7 g/dl (ce) no respiratory support: Hb <7.5 g/dl (ca); Hb <68 g/dl (ce) | Age: 1-7 days respiratory support: Hb <13.5 g/dl (ca); Hb <12.2 g/dl (ce) no respiratory support: Hb <12.0 g/dl (ca); Hb <10.9 g/dl (ce) Age: 8-14 days respiratory support: Hb <12.0 g/dl (ca); Hb <10.9 g/dl (ce) no respiratory support: Hb <10.0 g/dl (ca); Hb <9.0 g/dl (ce) Age: >15 days respiratory support: Hb <10.0 g/dl (ca); Hb <9.0 g/dl (ce) no respiratory support: Hb <8.5 g/dl (ca); Hb <7.7 g/dl (ce) |
| Widness et al., 2005 | Ventilation with ≥40% oxygen, sepsis, or necrotizing enterocolitis: Hct <40%  Ventilation with <40% oxygen or CPAP or hood treatment with ≥40% supplemental oxygen: Hct <35%  CPAP or hood oxygen: Hct <30%  Major surgical procedures: Hct <30% | Transfusion to maintain rational Hb level. |

RG, restrictive transfusion group; LG, liberal transfusion group; Hb, hemoglobin; Hct: hematocrit; CPAP, continuous positive airway pressure; ca, capillary; ce, central.
